# Integration of Biochar into Soil Unravels Protective Mechanisms Against Plastic-Induced Stress in *Lens culinaris* by Modulating Physiological Traits, Antioxidant Defense, and Methylglyoxal Detoxification Systems

**DOI:** 10.3390/plants15030470

**Published:** 2026-02-03

**Authors:** Riti Thapar Kapoor, Mirza Hasanuzzaman

**Affiliations:** 1 Centre for Plant and Environmental Biotechnology, Amity Institute of Biotechnology, Amity University Uttar Pradesh, Noida 201 313, Uttar Pradesh, India; rkapoor@amity.edu; 2 Department of Agronomy, Faculty of Agriculture, Sher-e-Bangla Agricultural University, Sher-e-Bangla Nagar, Dhaka 1207, Bangladesh; 3 Kyung Hee University, 26 Kyungheedae-ro, Dongdaemun-gu, Seoul 02447, Republic of Korea

**Keywords:** environmental pollutants, oxidative stress, plant water relations, xenobiotics

## Abstract

Plastics have emerged as a significant pollutant, posing a serious threat to the sustainability of the soil ecosystem and food security because of their long-term persistence, resilience, and robustness under different environmental conditions. The present investigation explored the impact of different doses of polypropylene (PP) on lentil plants and attenuation of the adverse impacts of PP by the application of pineapple fruit peel biochar (PBC). Lentil (*Lens culinaris*) plants exposed to PP treatment reduced morphological traits and relative water contents, reflecting photosynthetic injuries, a rise in lipid peroxidation, and electrolyte leakage. Utilization of PBC derived from waste biomass enhanced the growth attributes of lentils and alleviated PP-incited oxidative stress impacts. Polypropylene stress enhanced oxidative stress and increased enzymatic and non-enzymatic antioxidant variables in lentil plants. Antioxidant enzymes superoxide dismutase, catalase, ascorbate peroxidase, glutathione reductase, and glyoxalase enzymes were markedly upregulated in lentil after PBC amendment in PP3-treated soil. There was a significant reduction in methylglyoxal content by the activities of glyoxylase enzymes, minimizing the negative impacts of PP. Therefore, soil amendment with PBC protected lentil plants from PP-instigated oxidative disruption by modulating activities of antioxidant defense and glyoxalase system. Production of PBC from biomass wastes results in a safe, cost-effective, and ecofriendly material that can be used at the industrial level for the cultivation of crops in PP-contaminated soil. The novelty of the present research lies in promoting soil management practices and fostering our understanding of waste materials reutilization as renewable assets to combat the ecological implications of plastic pollution, and it emphasizes the treatment of plastic wastes with other waste materials and their practical applications to overcome plastic pollution.

## 1. Introduction

Plastics have become an inseparable part of modern society as they are widely used in household utensils, furniture, electronic goods, and packaging industries. The global plastic production was 400 million tons in 2022, and it is predicted to double by 2035 and almost quadruple by 2050 [1]. Plastics are artificially produced polymerized materials that are extensively used because of their low weight, thermostability, economical, non-corrosive, and flexible properties with high strength. Different types of plastics, such as polyethene terephthalate (PET), polypropylene (PP), polyvinyl chloride (PVC), polystyrene (PS), polylactic acid (PLA), etc., are widely used in different sectors. Recently, plastic contamination has become a major issue worldwide because of its prolonged persistence of up to thousands of years and robustness in different ecosystems. Plastics are omnipresent as they are reported in rivers, oceans, farmland, air, and polar regions [2]. Under different environmental conditions due to weathering, photodegradation, and oxidation, plastics undergo fragmentation and disintegrate into smaller pieces like macroplastics (>2.5 cm), microplastics (MPs; 0.1 μm–5 mm), and nanoplastics (NPs; <0.1 μm) [3]. The presence of additives in plastics, such as phthalates and bisphenol A, also known as endocrine disruptors, may cause cardiovascular diseases, cancer, kidney diseases, etc. [4,5]. Only 6–26% of plastic wastes are recycled, and the remaining 80–90% are discarded [6]. The soil has become a major sink for plastic contamination as plastic accumulation in terrestrial ecosystems is 4–23 times higher compared to aqueous systems, specifically in areas with more anthropogenic and agricultural activities [7]. Plastic particles can be absorbed by plant roots and move upwards through the vascular system, disrupt plant growth, and pose serious health risks as they enter animals and humans via the food chain [8].

In addition to direct entry of NPs/MPs in soil through improper disposal of plastic wastes in open areas, mulching of plastics, irrigation with wastewater, application of plastic-polluted sewage sludge, and organic fertilizers are the main sources of plastics in farming lands [9]. Utilization of sewage sludge as a soil amendment assists MP/NP entry into the soil matrix, as >90% plastics are found in sewage sludge [10]. Due to repetitive use of compost, plastic particles accumulate up to three million particles per hectare annually [11]. Plastic particles transfer from top to subsoil due to rainfall, tillage, irrigation, harvesting, etc. [12]. Persistent intrusion of plastics into the soil has been recognized as a major threat to food security and soil biodiversity [13]. After entering the soil, plastics can be considered as vectors for toxic metals/metalloids, organic contaminants, and agrochemicals, which may further aggravate the toxicity of the soil and adversely affect the health of plants [14]. The accretion of plastics in soil adversely affects physico-chemical properties of soil, water retention capacity, soil porosity and texture, soil bulk density, biogeochemical cycles, soil microbial diversity, soil flora and fauna, and plant development and potency [15]. Polyethylene MP exposure showed oxidative and DNA damage in *Arabidopsis thaliana* L. plants, which adversely affected their growth and development [16]. The chromosomal aberrations and DNA fragmentation were also reported in *Vicia faba* L. root tips, exhibiting genotoxic capacity of PS-MPs [17]. Plastic contamination reduced crop productivity by approximately 30% in onion, pea, cucumber, and rice yields [18].

Removing plastics from soil is a tedious task because of their minute size, wide distribution, and soil matrix complexity [19]. Polypropylene is widely used in packaging, textiles, medical devices, and the development of different consumer products due to its low cost and durability. Different procedures have been applied for plastic contaminants elimination from soil, such as catalysis, chemical oxidation/reduction processes, and adsorption. The adsorption is considered an effective, economical, and environmentally benign procedure. Biochar acts as a green material for the removal of plastics from soil as it alleviates the negative impacts of plastics on the properties of soil, plants, and microbial communities [20]. Wang et al. [21] and Elbasiouny et al. [22] observed that biochar application mitigated the adverse impacts of MPs. Biochar, as a sustainable biomass adsorbent, has several advantages, such as a unique structure with enhanced surface area, functional group availability, porosity, high durability and sorption capacity, and lower cost [23]. Biochar incorporation improves the properties of plastic-polluted soil; however, biochar properties depend on the temperature and the type of feedstock and its preparation procedures, etc. [24,25]. Lentil (*Lens culinaris* L.) has emerged as a sustainable alternative to animal-derived proteins, as they are rich in protein, essential amino acids, sugars, vitamins, minerals, dietary fiber, and functional phytochemicals [26,27]. Therefore, good agricultural practices for lentil production have come to the producers’ attention. Most of the studies have been conducted on the effect of MPs/NPs on the aquatic system [28]. Based on our information, for the first time, we have assessed the individual or combined effects of pineapple fruit peel biochar (PBC) and PP on the growth, antioxidant, and glyoxalase systems of lentil. The present study highlights the remediating potential of PBC used as a soil amendment, which would enhance growth and ameliorate biochemical variables of lentil plants growing under PP-contaminated soil. Hence, the transformation of agro-wastes into biochar and its incorporation into the soil provides sustainable solutions for waste management, boosting the circular economy using waste materials and preserving the environment for future generations.

## 2. Results

### 2.1. Proximate Analysis

Proximate estimation was conducted for the assessment of various components, such as moisture, ash, volatile matter, and fixed carbon contents present in the prepared biochar (PBC). The proximate analysis showed that PBC has 19.65% fixed carbon, 72.24% volatile material, 5.13% moisture, and 2.98% ash content.

### 2.2. Morphological Traits

The polypropylene exposure reduced root and shoot lengths and lentil plant biomass in comparison to control plants. Lentil plants showed a maximum reduction of 67%, 62%, 67%, and 84% in the length of roots and shoots and fresh and dry weight, respectively, after exposure to PP3 treatment over control (Table 1). However, PBC application increased root and shoot lengths by 26% and 20% and fresh and dry weight by 16% and 12%, respectively, over control plants. Higher doses of PP showed adverse impacts on the morphological traits of lentil plants with the following trend: PP3 > PP2 > PP1. The combined treatment PBC + PP3 resulted in 22% and 137% enhancement in fresh and dry weights, respectively, compared to PP3-treated plants. All the growth parameters were increased, but this was statistically significant only for the shoot length (*p* > 0.05). This may be due to high variability among replicates; thus, the differences did not reflect statistical significance, despite the visible trends. In contrast, shoot length showed less variability across replicates and was statistically significant. The PP1, PP2, and PP3 treatments reflected an 88%, 83%, and 62% reduction in the RWC, but the relative water content (RWC) was recorded as 95% with PBC, and it improved up to 83% with PBC + PP3 treatment in comparison to PP3-stressed plants (Table 1).

### 2.3. Pigment Content

Polypropylene treatment reduced chlorophyll (Chl) contents in lentil leaves. Maximum reductions of 47% and 29% in Chl and carotenoids (Car) amount were recorded in PP3 treatment compared to control plants (Table 2). However, the PBC amendment mitigated the negative effects of PP and significantly enhanced pigment content. A combined treatment (PBC + PP3) was effective and exhibited a 15% and 29% rise in Chl and Car over PP3-treated lentil. The maximum Chl (*a*/*b*) ratio was recorded in PP3-treated lentil leaves in comparison to the control.

### 2.4. Osmolyte and Phenolic Acid Contents

The application of PBC showed a remarkable rise in levels of different osmolytes, such as sugars, proline, glycine betaine, protein, and phenolic acid contents in lentil leaves. Lentil plants reflected a rise of 9%, 10%, 44%, and 59% in protein, sugar, glycine betaine, and proline contents, respectively, after PBC supplementation onto the soil compared to control lentil. Lentil plants grown under PP3 treatment exhibited a 128% and 86% rise in glycine betaine and proline contents, respectively, compared to control lentil (Table 3). The combined treatment of PBC and PP showed a rise in osmolytes in lentil compared to PP stress alone.

The PP1, PP2, and PP3 treatments showed negative impacts on protein contents, with a 17%, 34%, and 54% reduction over control lentil. However, the adverse effect was mitigated by PBC incorporation in soil, which reflected 8%, 16%, and 27% higher protein contents in comparison to lentil plants under PP1, PP2, and PP3 treatments, respectively. Polypropylene stress PP1, PP2, and PP3 caused a 13%, 27%, and 43% decrease in phenolic acid content in leaves in comparison to control. However, the PBC amendment significantly reversed this reduction. The highest rise of 92% in phenolic acid content was observed in combined PBC + PP3 treatment in comparison to lentil under PP3 stress alone.

### 2.5. Oxidative Stress Indicators

**Figure 1:**
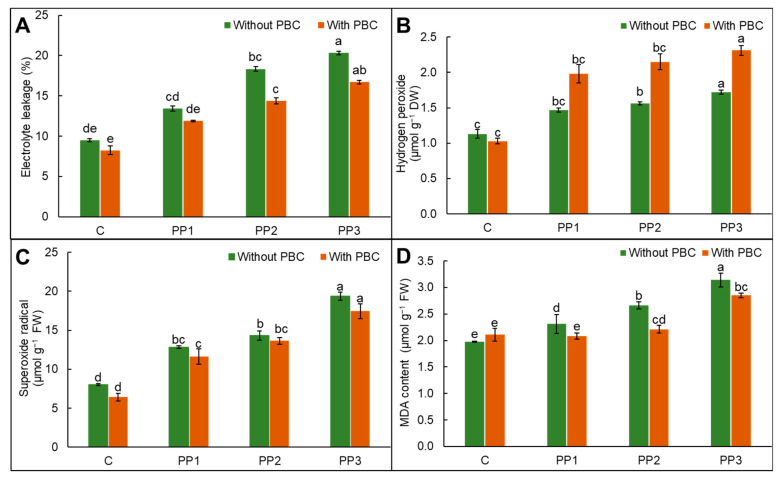
(**A**) Electrolyte leakage, (**B**) hydrogen peroxide, (**C**) superoxide content, and (**D**) malondialdehyde contents in *Lens culinaris* L. under polypropylene (PP) treatment with and without pineapple fruit peel biochar (PBC) as a soil amendment. Data are mean ± SD. Different letters on bars are significantly different at the *p* < 0.05 significance level as per Tukey’s honest significant difference (HSD) test.

Treatment with PP exhibited a rise in oxidative stress markers in comparison to untreated lentil plants. The rise in electrolyte leakage, hydrogen peroxide (H_2_O_2_), and superoxide radicles (O_2_^•−^) production was 114%, 52.21%, and 141.95%, respectively, with PP3 treatment over control lentil (Figure 1A–C). The addition of PBC in soil attenuated the adverse impacts of PP. Compared to PP1- and PP2-treated plants, PBC addition decreased electrolyte leakage by 12% and 22%, respectively. Amendment of PBC in PP3-treated pots significantly reduced the electrolyte leakage and O_2_^•−^ levels by 18% and 10%, respectively, over PP3 treatment, indicating the role of biochar in alleviation of oxidative stress.

Polypropylene treatment, i.e., PP1, PP2, and PP3, showed a 17%, 34, and 59% rise in MDA levels in comparison to untreated lentil plants. The soil incorporation with PBC combats the adverse impacts, as reflected with a reduction in malondialdehyde (MDA) contents of 10%, 17%, and 9%, respectively, in PBC + PP1, PBC + PP2, and PBC + PP3, respectively, in lentil in comparison to different doses of PP stress (Figure 1D). In the case of electrolyte leakage and H_2_O_2_ contents, high variability among replicates was recorded; thus, the differences did not reflect statistical significance, despite the visible trends. In contrast, MDA content showed comparatively less variability across replicates, thus showing statistical significance (*p* > 0.05).

### 2.6. Activities of Antioxidant Enzymes

**Figure 2:**
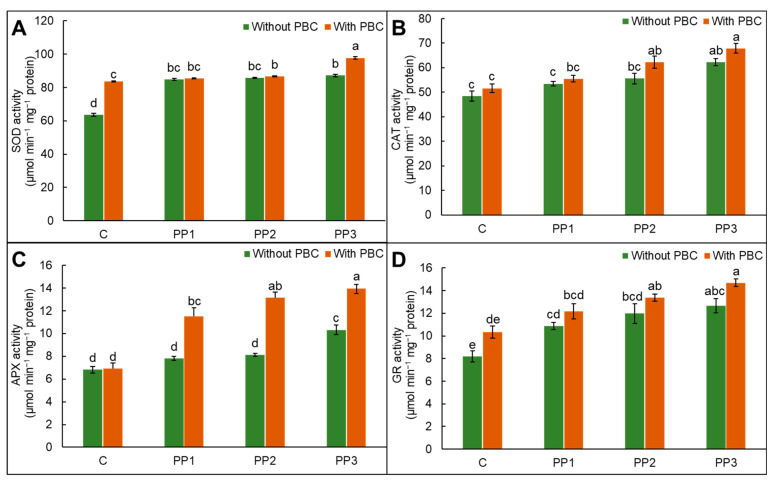
(**A**) Superoxide dismutase, (**B**) catalase, (**C**) ascorbate peroxidase, and (**D**) glutathione reductase in *Lens culinaris* L. grown under polypropylene (PP) stress with and without pineapple fruit peel biochar (PBC) as a soil amendment. Data are mean ± SD. Different letters on bars are significantly different at the *p* < 0.05 significance level as per Tukey’s honest significant difference (HSD) test.

The individual treatment or PBC addition in soil exhibited varied trends in superoxide dismutase (SOD), catalase (CAT), ascorbate peroxidase (APX), and glutathione reductase (GR) antioxidant enzyme activities. The supplementation of PBC enhanced antioxidant enzymes by 31%, 7%, 1%, and 27% for SOD, CAT, APX, and GR over control plants. Polypropylene treatment also increased antioxidative enzyme activities in comparison to control lentil. The amendment of PBC in PP3-treated soil resulted in a 12%, 9%, 35%, and 16% rise in SOD, CAT, APX, and GR activities, respectively, compared to lentil grown under PP3 treatment alone (Figure 2A–D). The results demonstrated that biochar can modulate the antioxidant system by enhancing SOD, CAT, APX, and GR activities and protect lentil under plastic stress.

### 2.7. Ascorbate–Glutathione Pool

**Figure 3:**
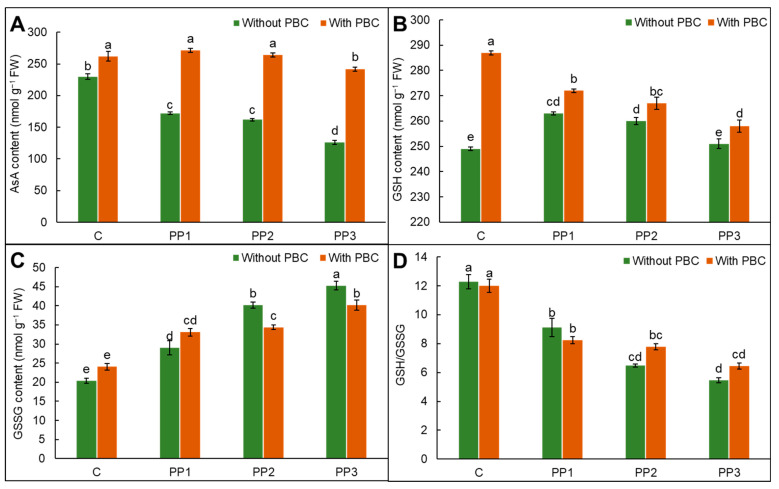
(**A**) Ascorbate, (**B**) GSH and GSSG (**C**) the GSH/GSSG ratio (**D**) in *Lens culinaris* L. grown under polypropylene (PP) treatment with and without pineapple fruit peel biochar (PBC) as a soil amendment. Data are mean ± SD. Different letters on bars are significantly different at the *p* < 0.05 significance level as per Tukey’s honest significant difference (HSD) test.

Polypropylene treatment without the incorporation of PBC decreased ascorbate (AsA) accretion but increased the glutathione (GSH) levels in lentil in comparison to control lentil. The PBC with PP application increased both AsA and GSH contents over PP-stressed lentil plants. The PBC treatment showed a 14% increase in AsA and a 15% enhancement in GSH in comparison to control lentil (Figure 3A,B). The PP3 treatment increased the glutathione disulfide (GSSG) amount up to 122% in comparison to control plants (Fig. 3C), showing a 55% decrease in the GSH/GSSG ratio. The PBC amendment in PP3-stressed lentil plants (PBC + PP3) mitigated the negative impacts of PP3 treatment and increased the GSH/GSSG ratio by up to 18% compared to the unamended plants with PP3 treatment (Figure 3D).

### 2.8. Glyoxalase Enzyme Activities and Methylglyoxal Content

**Figure 4:**
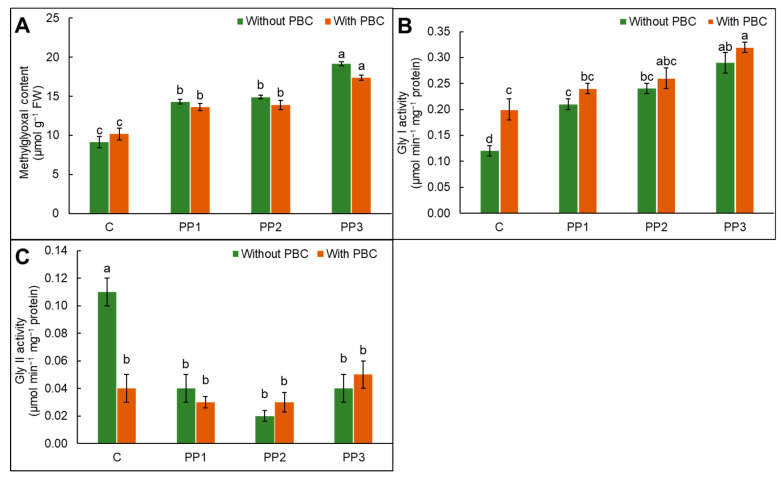
(**A**) Methylglyoxal, (**B**) glyoxalase I, and (**C**) glyoxalase II contents in *Lens culinaris* L. grown under polypropylene (PP) stress with and without pineapple fruit peel biochar (PBC) as a soil amendment. Data are mean ± SD. Different letters on bars are significantly different at the *p* < 0.05 significance level as per Tukey’s honest significant difference (HSD) test.

Exposure to PP3 treatment increased methylglyoxal content by up to 109% in comparison to control lentil plants. The PBC application, either alone or in combination, reduced methylglyoxal content in PP-stressed lentil, with a 9% reduction observed for PBC + PP3 over PP3 treatment (Figure 4A). Polypropylene treatment enhanced glyoxalase I (Gly I) and decreased Gly II activities over control lentil. A combined treatment of PBC + PP3 exhibited a 10% rise in Gly I activities in comparison to PP3-treatment (Figure 4B). Thus, PBC + PP3 addition showed a 150% increase in Gly II activity over PP3-treated lentil plants (Figure 4C). However, the difference was not statistically significant due to the variations among the replicates.

## 3. Discussion

Plant exposure to MPs/NPs induces oxidative stress, reduces plant growth, and adversely affects key physiological processes, ionic balance, photosynthesis, redox homeostasis, cytotoxicity, and genotoxicity in plant cells [29]. The MPs have rough surfaces and sharp edges, damage plant roots, and inhibit root growth [30]. Lentil seed germination was found to be sensitive to plastic stress [31]. In the present study, different doses of PP reduced the length of root and shoots, seedling weight, and RWC of lentils; however, the PBC amendment alleviated the adverse impact of PP treatments by enhancing moisture retention and microbial population in the soil. The decline in RWC in lentil leaves might be because of a decrease in hydraulic conductivity and cell turgor under PP stress. The amendment of PBC enhanced RWC, growth variables, and different biochemical parameters of lentil plants. Biochar promotes plant growth in plastic-contaminated soil due to its ability to regulate soil pH, improves physico-chemical properties of soil, and enhances nutrient availability and microbial abundance in the soil [32]. In addition, due to its porous structure and large surface area, biochar can easily adsorb microplastics from soil [33]. The non-polar plastic particles can stick to the hydrophobic surfaces of biochar via Van der Waals forces. The aromatic rings in biochar can form strong electronic linkages with aromatic plastics. The functional groups on the biochar can attract oppositely charged plastic particles, thus significantly mitigating plastic stress.

The negative impacts of plastics on the growth of plants and development depend on the type and concentration [34]. Exposure to plastics can cause genetic abnormalities and structural degeneration of plant cells by enhancing reactive oxygen species (ROS) generation through the disruption of gene expression and cell cycle [35]. In *Allium cepa* L. roots, PS-MPs caused a dose- and concentration-dependent decline in the mitotic index [36]. Plastic particles can bind to the surface of the roots of plants, impeding the pores of roots and hindering the uptake of water and nutrient absorption from soil. This blockage can lead to reduced root growth and trigger oxidative stress [37]. Plastic particles can infiltrate plants through their roots and can be transported in plants through both apoplastic and symplastic routes, reaching different parts of the plant [38]. However, plastic particles can also penetrate the xylem vessels through physical damage or cracks in the roots or stem and enter the plant cells [39]. Availability of surface functional groups like −NH_2_ and −SO_3_H on MPs adversely affect growth of plants [40]. The −NH_2_ functional group contains a positive charge, which can be easily adsorbed by the cell wall, thus inhibiting the adsorption of cationic elements by roots [41]. However, −SO_3_H, having negative charges, can combine with hydrophobic functional groups present in phospholipids of the cell membrane, as it can easily enter cells and show cytotoxicity in roots [42]. Plastics also showed cell atrophy and lignification in roots by decreasing root activity [40]. Due to MP stress, wheat and onion showed a reduction in growth and biomass accumulation [18]. The rate of seed germination in *Orychophragmus violaceus* L., *Impatiens balsamina* L., and *Trifolium repens* L. was significantly decreased due to PS supplementation [43].

Wang et al. [21] recorded that plastic particles are either stuck or trapped inside the biochar pores. The Van der Waals forces, electrostatic interactions, and chemical linkage between plastic particles and biochar regulate their retention mechanism [44]. Khalid et al. [6] reported that PVC inhibited the yield of crop plants and microbial population, whereas negative impacts were alleviated with biochar application. Therefore, biochar presence can buffer the adverse effects on soil microbes and their enzymatic activities by decreasing MP availability by promoting a conducive environment for plant growth.

Photosynthesis plays a significant role in plant development. Plastic stress-induced reduction in photosynthesis may be because of the generation of ROS, enhancing oxidative stress, changes in membrane proteins of photosystem II (PS II), chloroplast and thylakoid membrane peroxidation, changes in leaf ionome, and exfoliation of oxygen evolution complex, thus blocking electron transfer to PS II reaction centers, which adversely affect ETC reaction in PSII [40]. Application of PS-NPs reduced Chl contents and hindered photosynthesis in *Zea mays* L. and *Arabidopsis thaliana* L. [16]. The MP/NP presence on the leaf surface inhibits the opening of stomata, thus inhibiting photosynthesis [45]. In lettuce, MPs adversely affected various photosynthetic attributes, stomatal conductance, and Rubisco activity [46]. The present investigation confirmed a PP-incited reduction in Chl content, which might be because of decreased enzyme activity participating in the synthesis and peroxidation of chloroplast membranes, with fewer iron (Fe) and magnesium (Mg) ions accessible (Table 2). The higher Chl(*a/b*) ratio was observed under PP-treated lentil leaves, which may be due to lower production of Chl *b*. Similar findings were recorded by Mondal et al. [47]. There was a decrease in Car amount after treatment in lentil leaves with various doses of PP stress, reflecting decreased ability to scavenge free radicals. Ulhassan et al. [48] recorded that PS reduced Chl contents due to chloroplast structure degeneration. After reaching the leaves, plastic particles can accumulate within the mesophyll cells and disrupt leaf functioning, reducing photosynthetic efficacy, gas exchange, and plant development [49]. Chai et al. [50] showed that PVC-, PP-, and PE-induced reductions in the photosynthetic rate of mangrove were due to a reduction in Car and Chl *a* content and electron transport chain (ETC) efficiency. Application of PE resulted in the downregulation of >80% genes associated with PSI, PSII, and ETC in light-harvesting complexes in tobacco [51]. Biochar addition can promote the generation of Chl, electron transfer, and photosystem I and II activities in lentil plants [52].

Reactive oxygen species induce structural and functional damage to plant cells [16]. In the present study, we have reported a rise in H_2_O_2_ production, concomitantly with EL and lipid peroxidation in PP-stressed lentil; however, negative impacts were mitigated by PBC incorporation (Figure 1). Plastic stress resulted in H_2_O_2_ production in plant cells [53]. However, the increase in H_2_O_2_ content as observed in Figure 1B may be due to the dismutation of the superoxide radicals to H_2_O_2_, either spontaneously or through SOD activity, which enhanced H_2_O_2_ content. The H_2_O_2_ acts as a stable signalling molecule and may be involved in PP stress adaptation; thus, its increase does not necessarily indicate enhanced oxidative damage but a marker for PP stress resistance. Plants produce soluble sugars and proteins, which act as protectants under environmental stresses [37]. The sugar level assists plants in retaining energy, whereas protein acts as an osmotic regulator in plant cells, as it plays a prominent role in regulating the sustainability of intracellular enzymes. Sugars act as osmo-protectants and assist in ROS detoxification [54]. Biochar application improved the microbial community in plastic-contaminated soil, enhanced genes regulating carbohydrate and amino acid metabolism by assisting N and P metabolic pathways, and improved the growth of *Capsicum* plants [55]. Proline acts as an ROS scavenger and stress reliever, as its generation increases photosynthesis, stabilizes biomolecules, and maintains Na^+^ and K^+^ balance [56]. In lentil plants, sugar and proline accretion showed osmolyte involvement in combating PP pressure (Table 3). The phenolic compounds act as cell signaling molecules or antioxidants that assist in the production of secondary metabolites via malonic acid or the shikimic acid cycle [57]. Our results showed that PP treatment decreased phenol levels; however, PBC amendment in soil enhanced phenolic concentrations in PP-stressed lentil plants (Table 3). An increase in phenolic acid can decrease the negative impacts of PP, as it supports the plant defense system that can scavenge ROS production [58].

Enzymatic and non-enzymatic antioxidants present in plants enhance ROS removal [59]. The SOD and CAT function as the front line of defense to combat ROS. Polypropylene treatment increased antioxidative enzyme activities in comparison to control lentil (Figure 2A–D). Amendment of PBC significantly enhanced antioxidant enzymes by 31%, 7%, 1%, and 27% in SOD, CAT, APX, and GR over control lentil plants. *Oryza sativa* L. plants treated with plastic particles showed enhanced antioxidant enzymes, checking the negative implications of ROS [60]. The production of H_2_O_2_ is regulated by the ascorbate–glutathione pathway in plant cells. The AsA and GSH function as redox buffering agents that maintain redox balance and regulate water use efficacy, photosynthesis, the integrity of membranes, and metabolic pathways [61]. Enhancement in GSH and AsA in combined application exhibits defiance against PP toxicity for free radicals’ removal and redox homeostasis stability (Figure 3A,B). Glutathione reductase transforms GSSG to GSH to maintain the GSH/GSSG ratio for the regulation of physiological activities [62]. A rise in the activity of GR with PBC amendment enhanced ASA and GSH recycling to generate their reduced form, thus increasing the growth of lentil plants under PP-incited oxidative stress. In plant cells, methylglyoxal is produced via glycolysis; however, its excessive production inhibits the growth of cells and increases lipids and protein degradation [63]. Detoxification of methylglyoxal is catalyzed by glyoxalase I and II, which play a significant role in plants under environmental pressure [64]. Polypropylene stress enhanced glyoxalase I and reduced activities of Gly II (Figure 4B,C). Thus, the findings presented here underscore the fact that, in addition to enhancing ROS detoxification through modulation in antioxidant defenses and glyoxalase systems, soil amendment with PBC attenuates the disruptive effects of PP and regulates various physiological processes to overcome PP-incited oxidative pressure in lentil plants.

## 4. Materials and Method

### 4.1. Procurement of Chemicals and Seeds

Lentil (*Lens culinaris* L. variety KL-320) seeds were purchased from a local market. Polypropylene powder was purchased from Polymer Manufacturing Unit, Bawana, New Delhi, India. Polypropylene (molecular weight: 42.08 g mol^−1^) was a white-colored powder with 99% purity and used for experiments without any prior treatment or purification. Analytical-grade reagents were used for experiments, and these were purchased from Merck, Mumbai. All the solutions were prepared with deionized water under ambient conditions.

### 4.2. Preparation of Pineapple Fruit Peel Biochar

Pineapple fruit peels were collected from a local fruit vendor and rinsed, chopped into small pieces, and kept under sunlight for up to a week to reduce moisture amounts. For the preparation of PBC, a stainless-steel reactor was used, and nitrogen gas was used in a reactor to generate an inert environment. Dried pineapple fruit peel pieces were placed inside the reactor for 4 h at 400 °C, and then the prepared PBC was sieved to obtain a homogenous powder and used for pot experiments. Proximate analysis was conducted to determine various components present in the PBC.

### 4.3. Experimental Design

The sandy loam soil (pH 6.9) was used for experiments, and its electrical conductivity (EC) was 1.7 ds m^−1^. The pineapple fruit peel biochar and polypropylene powder were properly mixed in the soil as per the treatment. Optimal doses of PBC (2 g kg^−1^) and PP1, PP2, and PP3 (0.5 g kg^−1^, 1.0 g kg^−1^, and 1.5 g kg^−1^, respectively) were used in the present study. After proper mixing of the biochar or polypropylene powder in the soil, pots with treated soil were kept for 15 days in open air for thorough mixing of the incorporated materials. Lentil seeds were rinsed with 0.1% mercuric chloride (HgCl_2_) for 7 min and then cleaned properly with deionized water. The seeds were kept in a plastic pot (14 × 8 cm) with soil (600 g), and then the pots were kept in the growth chamber in 150 μmol photons m^−2^ s^−1^ photosynthetically active radiation (16 h light/8 h dark) at 25 ± 2 °C under 90% moisture. The following combinations were used in pot experiments: (i) control (containing soil without any treatment); (ii) PBC-treated (soil + 2 g kg^−1^ PBC); (iii) PP1 treatment (soil + 0.5 g kg^−1^ PP); (iv) PP2 treatment (soil + 1.0 g kg^−1^ PP); (v) PP3 treatment (soil +1.5 g kg^−1^ PP); (vi) PBC + PP1 treament (soil + 2 g kg^−1^ + 0.5 g kg^−1^ PP); (vii) PBC + PP2 treatment (soil + 2 g kg^−1^ + 1.0 g kg^−1^ PP); and (viii) PBC + PP3 treatment (soil + 2 g kg^−1^ + 1.5 g kg^−1^ PP). Pots were regularly irrigated with distilled water. The apical leaves of lentil (25-day-old plants) were used for the estimation of biochemical variables.

### 4.4. Measurement of Morphological Traits and Relative Water Content

Morphological traits were analyzed after removing lentil plants from the soil after washing the roots. Lentil seedling length and biomass were analyzed in both the control and treatment. The RWC was assessed by measuring lentil leaf disc weight. After that, the discs were soaked in autoclaved distilled water at 25 ± 2 °C for 12 h in the dark, and then the turgid weight was measured. To estimate the dry weight, discs were kept in an electric oven at 80 °C for 48 h, and the dry weight was estimated. The relative water content was calculated by the given equation:

(1) RWC (%) = [Fresh weight − Dry weight]/[Turgid weight − Dry weight] × 100

### 4.5. Measurement of Pigment Content

Pigment contents in lentil leaves were analyzed by macerating 0.5 g fresh leaves in 80% acetone [65]. At 647 and 663 nm, absorbance was measured to determine Chl *a* and *b*, respectively, with a spectrophotometer. Absorbance was taken at 470 nm for Car content. The following formulas were applied for the calculation of pigment contents:

(2) Chl *a* (mg g^−1^) = 12.25 × (A_663_) − 2.79 × (A_647_)

(3) Chl *b* (mg g^−1^) = 21.50 × (A_647_) − 5.10 × (A_663_)

(4) Total Chl (mg g^−1^) = (Chl *a*) + (Chl *b*)

(5) Car (mg g^−1^) = 1000 × (A_470_) − 1.82 × Chl *a* − 95.15 × Chl *b*/225

### 4.6. Assessment of Biochemical Parameters

#### 4.6.1. Sugar Content

Sugar content was analyzed with the Hedge and Hofreiter [66] procedure. Lentil leaves were kept in a water bath in 2.5 N HCl for up to 3 h at 100 °C. The Na_2_CO_3_ was mixed until effervescence was produced, and the reaction mixture was kept for cooling and was raised in volume up to 100 mL. In anthrone reagent (4 mL), the reaction mixture (1 mL) was incorporated, heated for 8 min, and then cooled. Optical density was measured at 620 nm, and sugar content was analyzed by a glucose standard curve.

#### 4.6.2. Proline

Proline was assessed by the Bates et al. [67] procedure. Lentil leaves (0.5 g) were kept in 3% sulfosalicylic acid, and then 2 mL leaf filtrate was mixed with glacial acetic acid (2 mL) and ninhydrin solution (2 mL), which was kept at 100 °C for 1 h and cooled. Toluene (4 mL) was mixed, and the upper layer was removed. At 520 nm, absorbance was measured. Proline content was calculated with a standard curve.

#### 4.6.3. Glycine Betaine

A total of 1 g of lentil leaves was kept in 10 mL of distilled water at 40 °C. Supernatant (1 mL) was added to 2 N HCl (1 mL), and the potassium iodide (KI) solution (0.2 mL), and the mixture was cooled for 90 min. Then, 1,2-dichloromethane (20 mL) and distilled water (2 mL) were incorporated and mixed at 40 °C. At 365 nm, the absorbance of the lower layer was measured by discarding the upper aqueous layer. The glycine betaine standard curve was used to estimate the glycine betaine amount.

#### 4.6.4. Protein

Leaves were crushed with 1 mL of 1 N NaOH and then placed at 100 °C for 5 min. The alkaline copper reagent was mixed and placed at room temperature for 15 min. The Folin–Ciocalteu reagent was added; after half an hour, absorbance was measured at 650 nm. The BSA standard curve was used to estimate protein content [68].

#### 4.6.5. Phenolic Acid Content

Lentil leaves (0.5 g) were crushed in 80% ethanol for 15 min and then centrifuged for 10 min at 10,000× *g*. In supernatant (3 mL), the Folin–Ciocalteu reagent (0.5 mL) and 20% Na_2_CO_3_ solution were mixed. Malick and Singh’s [69] method was used, and at 650 nm, optical density was analyzed. The gallic acid standard curve was used to estimate phenolic acid content.

### 4.7. Estimation of Oxidative Stress Indicators

#### 4.7.1. Electrolyte Leakage

Electrical conductivity (EC) was estimated by Dionisio-Sese and Tobita [70]. Electrolyte leakage (EL) was measured by the given formula:

(6) EL (%) = (EC_b_ − EC_a_) × 100/EC_c_

#### 4.7.2. Lipid Peroxidation

The Zhou and Leul [71] procedure was used to estimate lipid peroxidation by measuring MDA content using the following formula:

(7) MDA (nmol g^−1^ FW) = (OD_532_ − OD_600_) × A × V/a × E × W

Here, A, V, and a = volume of reaction solution; phosphate buffer, enzyme extract, and W = fresh weight; and E = MDA constant (1.55 × 10^−1^).

#### 4.7.3. Hydrogen Peroxide

Velikova et al.’s [72] procedure was used for H_2_O_2_ estimation. Lentil leaves (0.05 g) were fused in 0.1% trichloro acetic acid (TCA), and a 10 mM KH_2_PO_4_ buffer and a 1 M KI solution were added to a 0.5 mL extract. Optical density was measured at 390 nm, and H_2_O_2_ was analyzed by comparing the standard curve.

#### 4.7.4. Superoxide

Superoxide content was estimated by Yang et al. [73]. A total of 1 g of lentil leaves was macerated with 65 mM KH_2_PO_4_ buffer (pH 7.8) and then centrifuged at 5000× *g* for 10 min. In 1 mL filtrate, 1 mL each of 10 mM hydroxylamine hydrochloride, 17 mM sulfanilamide, and 97 mM naphthylamine were added prior to incubation at 25 °C for 20 min. At 530 nm, absorbance was calculated, and using a sodium nitrite standard curve, superoxide content was analyzed.

### 4.8. Assay of the Activities of Antioxidative Enzymes

Lentil leaves were macerated in a 0.1 M sodium phosphate buffer with polyvinylpyrrolidone (PVP). The mixture was centrifuged at 14,000× *g* for 30 min at 4 °C, and the filtrate was used to estimate enzymes.

#### 4.8.1. Superoxide Dismutase

Superoxide dismutase activity was analyzed by the Beyer Jr and Fridovich [74] method. The 2-nitrothiobenzoic acid (NBT) photoreduction was analyzed at 560 nm against the blank, and SOD activity was expressed as the enzyme amount required for a 50% decrease in NBT level.

#### 4.8.2. Catalase

The Aebi [75] method was applied for the analysis of the activity of catalase (CAT) by observing H_2_O_2_ degradation at 240 nm for 2 min. The CAT activity was assessed with an extinction coefficient of 39.4 mM^−1^ cm^−1^.

#### 4.8.3. Ascorbate Peroxidase

The 0.2 mL enzyme extract was mixed in a reaction mixture (2 mL) made up of 0.1 mM EDTA, 25 mM potassium phosphate buffer, 1 mM H_2_O_2_, and 0.25 mM AsA. Absorbance was measured at 290 nm for 60 s, and APX activity was measured with an extinction coefficient of 2.8 mM^−1^ cm^−1^ with the Nakano and Asada [76] method.

#### 4.8.4. Glutathione Reductase

The Foster and Hess [77] procedure was used to analyze the change in optical density at 340 nm for 3 min. A 3 mL reaction mixture was prepared from 1 mM EDTA, 50 μM NADPH, 100 mM potassium phosphate buffer, 100 μM GSSG, and 100 μL enzyme, and GR activity was expressed as µmol min^−1^ mg^−1^ protein.

### 4.9. Determination of Ascorbate and Glutathione Pool

Leaves of lentil were kept in 6% TCA, and 2% dinitrophenylhydrazine containing 10% thiourea was added to the supernatant. Then, they were heated for 15 min, cooled, and mixed with 80% H_2_SO_4_. Absorbance was calculated at 530 nm, and ascorbate (AsA) content was estimated with a standard curve of AsA [78]. The Ellman [79] procedure was used for GSH estimation. Absorbance was measured at 412 nm, and the GSH amount was assessed with a GSH standard curve. The GSH amount analyzes changes in absorption rate during the production of NBT from 5,5-dithio-bis (2-nitrobenzoic acid) (DTNB) reduction. The GSSG was calculated after GSH elimination by 2-vinylpyridine derivatization. The GSH amount was analyzed by reducing GSSG from total GSH, and the GSH/GSSG ratio was assessed by Hasanuzzaman et al.’s [80] procedure.

### 4.10. Measurement of Methylglyoxal Content

A total of 1 g of leaves was fused with 5 mL of 0.5 M perchloric acid and centrifuged at 11,000× *g*. Charcoal (10 mg mL^−1^) was mixed in the filtrate for decolorization, and after centrifugation, the supernatant was neutralized by K_2_CO_3_ solution, and 500 mM N-acetyl-l-cysteine (20 mL) was added in neutralized filtrate (980 mL) and placed at 22 °C for 5 min. Then, N-α-acetyl-S-(1-hydroxy-2-oxoprop-1-yl) cysteine synthesis was estimated at 288 nm by the Wild et al. [81] method.

### 4.11. Assay of Glyoxalase Enzyme Activities

The mixture (700 µL) included a leaf (0.5 g) extract made up of a 100 mM K_2_HPO_4_ buffer, 1.7 mM GSH, 3.5 mM methylglyoxal, and 15 mM MgSO_4_. The absorbance was read at 240 nm for 60 s, and at 3.37 mM^−1^ cm^−1^, an extinction coefficient was applied to analyze Gly I activity [48]. The reaction mixture (1 mL) was composed of 0.5 g lentil leaf extract, 100 mM Tris-HCl buffer, 0.2 mM DTNB, and 1 mM S-d-lactoylglutathione. The Principato et al. [82] method was used for Gly II activity calculation with a 13.6 mM^−1^ cm^−1^ extinction coefficient.

### 4.12. Statistical Analysis

The experiments were performed in a randomized block design with three replications. The data obtained were analyzed using c and Tukey’s honest significant difference (HSD) test.

## 5. Conclusions

This study highlights the utilization of PBC agro-wastes as an ecofriendly, cost-effective, and feasible approach that promotes remediation of soil and waste management concomitantly. The PP-incited stress decreased RWC and growth of lentil plants, whereas it enhanced oxidative stress indicator levels like electrolyte leakage, MDA, H_2_O_2,_ and O_2_^•−^. The addition of PBC in PP-treated soil increased growth variables and chlorophyll amount, whereas it reduced markers of oxidative stress in PP-treated lentil plants. Synergistic treatment of PBC and PP enhanced antioxidant enzyme activities and, under PP stress, regulated high AsA and GSH contents in lentil plants. Marked accretion of osmolytes was detected in lentil leaves in response to soil amendment with PBC. The synergistic application (PBC + PP) increased Gly I and II activities to promote detoxification of methylglyoxal. However, the present study has been conducted under laboratory conditions to analyze the effect of one type of biochar, i.e., PBC, on the mitigation of PP. Thus, further investigations are needed to elucidate the impact of different types of biochar derived from agricultural wastes on the various types of plastics present in the soil, dose optimization, mode of application, PP absorption mechanism in plants, and PBC function in enhancing the growth of plants under multiple environmental pressures for its practical applications under field conditions. There is an urgent need for soil protection from plastic contamination, not only for maintaining resilient agricultural practices but also for food security towards achieving sustainable development goals. The findings of the present investigation clearly reveal that agricultural waste materials, such as PBC, can be used as green adsorbents, which not only save the environment but also boost a closed-loop economy that would lead to a sustainable future.

## Figures and Tables

**Table 1:** Length of seedlings, biomass, and relative water content in *Lens culinaris* L. treated with polypropylene (PP) with or without pineapple fruit peel biochar (PBC) as soil amendment. Data are mean ± SD. Different letters on columns are significantly different at the *p* < 0.05 significance level as per Tukey’s honest significant difference (HSD) test.

Treatment	Root Length (cm)	Shoot Length (cm)	Fresh Weight (g)	Dry Weight (g)	Relative Water Content (%)
C	11.86 ± 0.17 ^b^	21.38 ± 0.94 ^b^	9.39 ± 0.09 ^bc^	2.77 ± 0.16 ^a^	91.94 ± 0.18 ^b^
PBC	14.92 ± 0.04 ^a^	25.69 ± 0.3 ^a^	10.89 ± 0.39 ^a^	3.1 ± 0.14 ^a^	95.08 ± 0.46 ^a^
PP1	9.29 ± 0.28 ^c^	16.92 ± 0.34 ^c^	9.37 ± 0.29 ^bc^	2.43 ± 0.11 ^ab^	87. 96 ± 0.29 ^c^
PP2	6.17 ± 0.57 ^de^	11.36 ± 0.39 ^e^	7.63 ± 0.32 ^d^	1.4 ± 0.25 ^c^	83.18 ± 0.13 ^d^
PP3	3.72 ± 0.39 ^f^	8.04 ± 0.17 ^f^	3.13 ± 0.18 ^e^	0.45 ± 0.16 ^d^	62.35 ± 0.33 ^e^
PBC + PP1	9.95 ± 0.53 ^c^	18.34 ± 0.41 ^c^	10.03 ± 0.49 ^ab^	2.67 ± 0.18 ^a^	90.53 ± 0.44 ^b^
PBC + PP2	7.12 ± 0.14 ^d^	13.98 ± 0.36 ^d^	8.2 ± 0.19 ^cd^	1.74 ± 0.27 ^bc^	87.77 ± 0.38 ^c^
PBC + PP3	5.02 ± 0.38 ^ef^	9.86 ± 0.19 ^e^	3.81 ± 0.26 ^e^	1.07 ± 0.05 ^cd^	83.17 ± 0.16 ^d^

**Table 2:** Effect of polypropylene (PP) treatment on pigment contents of *Lens culinaris* L. with or without pineapple fruit peel biochar (PBC) as soil amendment. Data presented are mean ± SD. Different letters on columns are significantly different at the *p* < 0.05 significance level as per Tukey’s honest significant difference (HSD) test.

Treatment	Chlorophyll *a* (mg g^−1^ FW)	Chlorophyll *b* (mg g^−1^ FW)	Chlorophyll (*a + b*) (mg g^−1^ FW)	Chlorophyll *a*/*b*	Carotenoids (mg g^−1^ FW)
C	1.54 ± 0.02 ^b^	0.78 ± 0.02 ^a^	2.32 ± 0.01 ^b^	1.97 ± 0.01	0.48 ± 0.02 ^abc^
PBC	1.72 ± 0.01 ^a^	0.82 ± 0.01 ^a^	2.54 ± 0.01 ^a^	2.08 ± 0.07	0.57 ± 0.03 ^a^
PP1	1.48 ± 0.02 ^b^	0.45 ± 0.07 ^bc^	1.93 ± 0.04 ^cd^	3.29 ± 0.21	0.46 ± 0.01 ^bc^
PP2	1.28 ± 0.01 ^c^	0.33 ± 0.03 ^cd^	1.61 ± 0.05 ^ef^	3.88 ± 0.27	0.41 ± 0.01 ^cd^
PP3	1.04 ± 0.03 ^e^	0.19 ± 0.02 ^d^	1.23 ± 0.04 ^g^	5.47 ± 0.56	0.34 ± 0.01 ^d^
PBC + PP1	1.55 ± 0.03 ^b^	0.58 ± 0.02 ^b^	2.13 ± 0.03 ^bc^	2.67 ± 0.37	0.52 ± 0.02 ^ab^
PBC + PP2	1.34 ± 0.04 ^c^	0.46 ± 0.09 ^bc^	1.81 ± 0.11 ^de^	2.91 ± 0.07	0.48 ± 0.03 ^abc^
PBC + PP3	1.16 ± 0.03 ^d^	0.25 ± 0.01 ^d^	1.42 ± 0.03 ^fg^	4.64 ± 0.34	0.44 ± 0.02 ^bc^

**Table 3:** Effect of polypropylene (PP) on osmolytes and phenolic acid contents of *Lens culinaris* L. with or without pineapple fruit peel biochar (PBC) as soil amendment. Data are mean ± SD. Different letters on columns are significantly different at the *p* < 0.05 significance level according to Tukey’s honest significant difference (HSD) test.

Treatment	Sugar (mg g^−1^ DW)	Proline (µM g^−1^ DW)	Glycine Betaine (µg g^−1^ DW)	Protein (mg g^−1^ FW)	Phenolic Acid Content (mg g^−1^ FW)
C	4.97 ± 0.09 ^ab^	14.25 ± 0.20 ^e^	2.50 ± 0.07 ^f^	18.05 ± 0.08 ^b^	7.17 ± 0.22 ^bc^
PBC	5.49 ± 0.19 ^a^	22.63 ± 1.01 ^d^	3.60 ± 0.21 ^e^	19.59 ± 0.27 ^a^	8.6 ± 0.19 ^a^
PP1	4.18 ± 0.16 ^c^	23.54 ± 0.61 ^d^	4.29 ± 0.13 ^d^	14.94 ± 0.51 ^cd^	6.23 ± 0.11 ^d^
PP2	3.61 ± 0.24 ^cd^	24.64 ± 0.94 ^cd^	4.9 ± 0.11 ^d^	11.86 ± 0.45 ^e^	5.23 ± 0.10 ^e^
PP3	2.55 ± 0.17 ^e^	26.45 ± 0.75 ^c^	5.69 ± 0.06 ^c^	8.25 ± 0.22 ^f^	4.06 ± 0.16 ^f^
PBC + PP1	4.86 ± 0.15 ^b^	40.6 ± 0.73 ^b^	6.86 ± 0.33 ^b^	16.16 ± 0.11 ^c^	6.53 ± 0.23 ^cd^
PBC + PP2	4.16 ± 0.04 ^c^	44.52 ± 0.60 ^a^	7.75 ± 0.17 ^a^	13.72 ± 0.39 ^d^	7.00 ± 0.19 ^c^
PBC + PP3	3.39 ± 0.09 ^d^	46.35 ± 0.34 ^a^	8.35 ± 0.09 ^a^	10.49 ± 0.49 ^e^	7.81 ± 0.21 ^b^

## Data Availability

All data are available in this article.

## Abbreviations

The following abbreviations are used in this manuscript:

APX	Ascorbate peroxidase
CAT	Catalase
MDA	Malondialdehyde
GR	Glutathione reductase
GSH	Reduced glutathione
GSSG	Oxidized glutathione
PBC	Pineapple fruit peel biochar
PP	Polypropylene
ROS	Reactive oxygen species
RWC	Relative water content
SOD	Superoxide dismutase
